# Artificial intelligence for coordinating vaccine design, antiviral discovery, and real-world monitoring in the era of emerging and endemic viral threats

**DOI:** 10.3389/fphar.2026.1773609

**Published:** 2026-05-08

**Authors:** Adewunmi Akingbola, Abiodun Adegbesan, Olajumoke Adewole, Kehinde O. Adebiyi, Akpevwe Emmanuel Benson, Olajide Ojo, Jessica Otumara Urowoli, Opeyemi Joshua Alabi, Oluwaremilekun Juliet Ojeriakhi, Mayowa Shekoni

**Affiliations:** 1 Department of Public Health and Primary Care, University of Cambridge, Cambridge, United Kingdom; 2 Department of Global Health, African Cancer Institute, Stellenbosch University, Cape Town, South Africa; 3 Department of Biology, Indiana University, Bloomington, IN, United States; 4 Faculty of pharmacy, University of Benin, Benin City, Nigeria; 5 School of Applied Sciences, University of West of England, Bristol, United Kingdom; 6 School of Allied Health Sciences, Anglia Ruskin University, Cambridge, United Kingdom; 7 Department of Community Health and Primary Care, Lagos State University College of Medicine, Ojo, Nigeria; 8 Department of Public Health, John Hopkins University, Baltimore, MA, United States; 9 Department of Community Health, Lagos State University College of Medicine, Ojo, Nigeria

**Keywords:** artificial intelligence, global health preparedness, infectious diseases, machine learning, pandemics, vaccine design

## Abstract

Vaccine development has traditionally been a lengthy and resource-intensive process, often struggling to keep pace with rapidly emerging infectious threats. Recent advances in artificial intelligence (AI) offer new opportunities to transform this landscape by enabling faster, more precise, and data-driven approaches to vaccine design. This review examines how AI is being applied across key stages of vaccine development, including antigen discovery, epitope prediction, structural optimisation, immunogenicity assessment, and safety evaluation. We highlight the use of machine learning and deep learning models to analyse large-scale genomic, proteomic, and immunological datasets, allowing for more targeted identification of promising vaccine candidates. While AI-driven approaches show considerable promise, their successful translation into effective vaccines depends on the quality and representativeness of the data used, as well as close integration with experimental and clinical validation. Current challenges include data bias, limited representation of populations from low- and middle-income countries, and the need for transparent and interpretable models that can support regulatory decision-making. By synthesising recent developments and ongoing challenges, this review underscores the potential of AI to complement traditional vaccine development pipelines. When responsibly implemented, AI-based methods may accelerate vaccine innovation, improve global preparedness, and support more equitable responses to future infectious disease outbreaks.

## 1 Introduction

The most recent global viral pandemic, the coronavirus disease 2019 (COVID-19) pandemic, has exposed a long list of notable gaps in the preparedness of global public health to viral outbreaks, gaps which have existed for many years but are now more pronounced in the areas of spread and the suffering caused by this emerging viral infection (Schwartz, 2021). One other thing COVID-19, as an example, highlighted as a weakness of global public health is the fact that these lethal viral pathogens do not in any way respect the borders of nations; having a well-constructed border is not enough for prevention, proper public health measures must be put in place to ensure adequate prevention (Schwartz, 2021).

An emerging infectious disease, as defined by the Centres for Disease Control and Prevention (CDC) simply means “infectious diseases whose incidence in humans has increased in the past 2 decades or threatens to increase in the near future” (Centers for Disease Control and Prevention, 2026). Other emerging viruses include, Borealpox (Ukoaka et al., 2024), Nipah virus (Ukoaka et al., 2024), Oropuche virus (Akingbola et al., 2024), Hendra virus (Ukoaka et al., 2024). The effect of these emerging viral threats on global health is now more pronounced and widespread, affecting societies, public health and economies (Oluwanifemi Akomolafe et al., 2024). These viruses, as seen with the COVID-19 pandemic can overwhelm healthcare systems, eventually increasing morbidity, mortality and ultimately, healthcare facilities worldwide faced challenges, which includes limited personnel and medical supplies (Schwartz, 2021; Oluwanifemi Akomolafe et al., 2024). The ripple effect of the stretch on healthcare resources affected the treatment of COVID-19 patients and altered routine medical care, worsening outcomes for other health conditions (Oluwanifemi Akomolafe et al., 2024; Weissglass, 2022). The global nature of emerging viral threats necessitates coordinated international responses and an effective surveillance, quick capacity for diagnosis, and an extensive healthcare infrastructure are crucial for managing these threats (Oluwanifemi Akomolafe et al., 2024). In addition, there is a need for global collaboration in research and development, as observed in the quick production and distribution of the COVID-19 vaccines, which is vital for developing and deploying effective antiviral strategies (Oluwanifemi Akomolafe et al., 2024). Furthermore, among all of the groups of emerging infectious diseases, emergent viral agents remain the most severe threat to global health and populations as they are caused by a highly heterogeneous group of agents which includes previously undetected or unknown viruses; previously known viruses whose causative role in causing clinical disease has previously not been identified; evolution of new genetic strains from initially identified viral agents; and known viruses that have spread to new geographic areas or to new populations (Schwartz, 2021). Those viral agents whose role in causing human disease had significantly declined in the past but have once again become prevalent as a cause of illness, are termed reemerging viral diseases (Schwartz, 2021).

Irrespective of the efficacy of traditional antiviral agents, many of these viruses can develop resistance through a couple of mechanisms, and this poses a significant challenge to antiviral therapy (Oluwanifemi Akomolafe et al., 2024; Iyede et al., 2023). Resistance occurs due to the increased mutation rates of viruses, more specifically RNA viruses, due to its absent proofreading mechanisms during replication (Joseph et al., 2022). This in turn leads to the quick emergence of viral variants with mutations that confer resistance to a wide range of antiviral drugs (Oluwanifemi Akomolafe et al., 2024). A common mechanism of resistance is viral enzyme mutation or mutation of proteins targeted by antiviral drugs (Iyede et al., 2023). Examples are the mutations in the HIV reverse transcriptase or protease enzymes (Oluwanifemi Akomolafe et al., 2024; Iyede et al., 2023) and influenza virus neuraminidase enzyme (Oluwanifemi Akomolafe et al., 2024; Schalkwijk et al., 2022). Some antiviral drugs require activation by host or viral enzymes to become effective (Joseph et al., 2022). Mutations that alter this activation pathways can confer resistance (Oluwanifemi Akomolafe et al., 2024; Iyede et al., 2023). For example, some herpes simplex virus thymidine kinase enzyme mutation (Oluwanifemi Akomolafe et al., 2024; Iyede et al., 2023; Joseph et al., 2022; Schalkwijk et al., 2022).

Artificial Intelligence (AI) has become a force in healthcare, particularly in antiviral research, by using tools like neural networks and deep learning to solve complex challenges and drive innovation (Lopez-Rincon et al., 2021). In medicine, AI and machine learning (ML) are revolutionizing antiviral drug discovery by analyzing large biological datasets to identify potential candidates faster than traditional methods (Oluwanifemi Akomolafe et al., 2024). ML models, trained on existing antiviral compounds and viral structures, can predict the activity of new molecules, significantly speeding up the process. A key example is during the COVID-19 pandemic, when AI helped identify potential inhibitors of the SARS-CoV-2 main protease (Oluwanifemi Akomolafe et al., 2024; Khakharia et al., 2021), shortening the drug discovery timeline and enabling quicker responses to emerging viral threats (Oluwanifemi Akomolafe et al., 2024; Zeng et al., 2021). Beyond drug development, AI is also vital for real-time surveillance and population monitoring, integrating data models, mobile location data, travel information, and epidemiological patterns to improve early detection and response (Lopez-Rincon et al., 2021; Khakharia et al., 2021; Zeng et al., 2021; Khan et al., 2012). One notable application is the Infectious Disease Vulnerability Index (IDVI), which was used during the COVID-19 outbreak to assess country-level vulnerability, incorporating factors like healthcare capacity and socio-economic conditions (Ong et al., 2021; npj, 2025; Vita et al., 2015; Martini et al., 2020). Combining population-level assessments with individual data can enhance AI-driven risk prediction, supporting targeted public health interventions (Lundegaard et al., 2008).

Importantly, the increasing frequency of emerging and reemerging viral diseases, together with their rapid evolution and growing resistance to existing antiviral therapies, has exposed fundamental limitations in current biomedical and public health preparedness strategies. While artificial intelligence has been applied independently to antiviral discovery, vaccine design, and epidemiological surveillance, these efforts remain largely siloed, limiting their translational and real-world impact. Thus, there is a critical need for an integrated framework that connects AI-driven molecular discovery with real-time surveillance and public health implementation. This review addresses this gap by synthesizing existing AI approaches across interrelated domains to define a unified preparedness architecture that integrates algorithmic innovation, biomedical data integration, and population-level monitoring. The review outlines an integrative framework in which AI accelerates vaccine and antiviral development and enables coordinated, adaptive, and evidence-based responses to both emerging and endemic viral threats, by examining convergences between AI tool design, data-driven discovery, and public health implementation.

## 2 Literature search strategy

This narrative review was informed by a structured search of relevant literature to identify studies and tools related to artificial intelligence applications in vaccine development. Searches were conducted in PubMed, Scopus, Web of Science, and Google Scholar. Key search terms included combinations of *“artificial intelligence,” “machine learning,” “deep learning,” “vaccine development,” “vaccine design,” “pandemic preparedness”* and *“computational vaccinology.”* The search focused on publications from 2010 till date to capture recent advances in AI-driven vaccine research. Studies were included if they described AI-based approaches, computational tools, or platforms applied to vaccine discovery, antigen prediction, or vaccine optimization. Articles not focused on vaccine-related applications, non-English publications, and opinion pieces without methodological descriptions were excluded. In addition to peer-reviewed publications, grey literature sources, including official tool or platform websites and technical documentation, were consulted to identify relevant computational tools and obtain updated information on their functionality and availability. Tools discussed in the review were selected based on reported use in the literature, accessibility, and evidence of validation or demonstrated application in vaccine research.

## 3 Existing AI tools in vaccine design and optimization

### 3.1 Current landscape of AI-assisted vaccine platforms

In today’s landscape, AI-assisted vaccine platforms encompass a connected set of capabilities that span the vaccine design and update cycle, including antigen target triage, epitope mapping, immunogenicity scoring, and forecasting of variant emergency (Zhao and Zai, 2025). In practice, most deployments are not end-to-end AI-designed vaccines, but decision support systems that compress the search space and help prioritize candidates for testing. The field has matured quickly over the past 5 years because outbreak timelines, rapidly expanding sequence surveillance, and growth in immunopeptidomics have made it both necessary and feasible to use models for prioritization rather than exhaustive experimentation (Olawade et al., 2024; Elfatimi et al., 2025). In parallel, AI and ML approaches are increasingly interwoven with the wider computational toolkit used in vaccine development, including adjuvant screening, immunogen design, docking, and molecular dynamics simulation. Thus, the use of AI in the vaccine development pipeline is realistically a stack of interoperating tools rather than a single model.

Firstly, in antigen prediction and target triage, most platforms often begin with proteome-scale screening to decide which viral proteins or regions are most promising as vaccine targets (Lopez-Rincon et al., 2021). Reverse vaccinology workflows use sequence-derived features and learned rankings to propose candidate antigens, sometimes extending beyond canonical surface proteins to include conserved non-structural targets (Ong et al., 2021).

Further, epitope mapping is arguably the most established and widely adopted layer of AI use in vaccine pipeline development, largely due to the richness of available data (npj, 2025). A central community resource is the Immune Epitope Database and Analysis Resource, which curates a very large catalogue of experimentally described immune epitopes across pathogens, autoimmune conditions, and cancer targets, and provides analysis tools that support vaccine design decisions (Vita et al., 2015). In practical workflows, this resource is used to retrieve known epitopes, benchmark predictions, and run integrated analyses, including population coverage, epitope conservation, and clustering, to focus attention on high-value candidate regions (Martini et al., 2020). Flowing from this, widely used MHC binding and presentation predictors, such as the NetMHC family developed at the Technical University of Denmark, provide computational estimates of peptide-MHC interactions that are foundational for T-cell vaccine design (Lundegaard et al., 2008). For example, T-cell epitope prediction tools integrate MHC class I and II binding predictions by estimating IC50 values for peptides binding to specific MHC molecules, alongside peptide-processing predictions that account for intracellular antigen processing, and immunogenicity prediction models that help identify peptide-MHC complexes likely to elicit immune responses (Bioinformatics, 2025).

Moving beyond epitope nomination, immunogenicity scoring is another approach being used in ML model development for vaccine design. This aims to estimate whether a presented peptide is likely to elicit a functional immune response. However, platforms are still constrained by noisy labels, assay variability, and host-dependent effects such as immune history and TCR repertoire differences. As a result, immunogenicity tools are best treated as ranking systems that enrich for likely responders rather than definitive classifiers (T-SCAPE, 2025).

More so, in recent times, there has been a rapid adoption of structure-aware analysis and interaction modelling. Docking software supports vaccine research by estimating the interaction geometry and energetics between proteins, or between peptides and their binding partners, helping evaluate whether proposed epitopes or immunogens are structurally plausible and likely to engage immune receptors (Muhammad, 2021). RosettaDock is widely used for modelling protein–protein interactions by searching rigid-body and side-chain conformational space to identify low-energy complexes (Lyskov and Gray, 2008). PyRosetta, which provides programmatic access to Rosetta modelling, is similarly used for protein structure prediction and design, and can support vaccine development by enabling iterative optimisation of candidate immunogens, structural refinement, and evaluation of peptide-MHC interaction hypotheses at a structural level (Ford et al., 2020). Substantiating this, the development of AlphaFold by Google DeepMind has not only revolutionized biology and medicine but has also significantly impacted vaccine design frameworks (Jumper et al., 2021).

In parallel, general-purpose computational biology libraries and machine learning frameworks support the day-to-day implementation of vaccine pipelines. BioPython is frequently used for sequence handling, feature extraction, and pipeline orchestration, and it can be integrated with structure modelling tools to support end-to-end workflows from sequence analysis to structural evaluation (Olawade et al., 2024). On the machine learning side, TensorFlow and similar frameworks underpin many bespoke models for peptide-MHC binding, protein-protein interaction prediction, and large-scale sequence representation learning, and they enable rapid training and deployment of models that learn from genomic, proteomic, and immunological datasets (Ford et al., 2020).

Also, variant emergence forecasting and strain or variant selection are becoming increasingly important, particularly for rapidly evolving viruses and recurring vaccination cycles (MDPI, 2025; Communications Medicine, 2025). In this case, AI models integrate viral evolution, antigenic change, and epidemiological dominance signals to help anticipate which lineages are likely to dominate and which antigenic drift patterns may erode protection, thus aiding major health decisions, as in the case of influenza, where strain updates are time-limited and must be made under uncertainty (Kraemer et al., 2025).

Overall, the current landscape is best understood as a modular platform ecosystem rather than a single approach. Epitope prediction and population coverage tools help provide breadth and speed, while immunogenicity scoring adds a necessary filter for functional relevance, and structural modelling and docking bring mechanistic constraints, with forecasting tools helping to connect design to viral evolution.

### 3.2 Evidence from real-world deployments of AI tools in vaccine design

The use of AI tools in vaccine technology has seen multiple pragmatic applications over the past decade, with a notable boom in the last 5 years, driven largely by the COVID-19 pandemic and the recent broader adoption of AI and the associated push to incorporate these methods across relevant fields (Jirui, 2025; Ghosh et al., 2023; Gawande et al., 2025). Consequently, AI has been deployed across multiple points in modern vaccinology pipelines, including prioritizing vaccine targets from sequence data (reverse vaccinology), predicting T- and B-cell epitopes and population coverage, designing antigens and immunogens, and forecasting viral evolution to support strain/variant selection (El Arab et al., 2025; Bravi, 2024). While there are numerous direct uses of AI in vaccine design, here we spotlight how AI tools are redefining vaccine development in selected viral pipelines.

### 3.3 SARS-CoV-2

The use of AI in SARS-CoV-2 vaccine design spans rapid antigen/epitope prioritization, peptide set optimization, and immune-escape-aware antigen design. Specifically, early in the COVID-19 pandemic, reverse vaccinology platforms enhanced with machine learning were used to triage SARS-CoV-2 proteins beyond the spike antigen. For example, Vaxign/Vaxign-ML was applied to the SARS-CoV-2 proteome to prioritize candidate antigens (including spike and selected non-structural proteins) through predictive features linked to protective immunity and feasibility for vaccine development (Ong et al., 2021). This is also reflected in epitope prediction and peptide vaccine formulation, where AI-enabled immunoinformatics tools have been used to prioritize T-cell epitopes, evaluate population coverage, and assemble candidate epitope sets. In parallel, deep learning-driven immunoinformatics pipelines such as DeepVacPred and related multi-epitope design workflows were used to nominate SARS-CoV-2 epitope combinations as candidate vaccine constructs for subsequent experimental evaluation (Muhammad, 2021; Immunity, 2025). More recently, SARS-CoV-2 has served as a proving ground for AI models that explicitly anticipate immune escape. The EVEscape framework used a combination of generative learning and evolutionary reasoning to estimate escape potential and to help prioritize mutations likely to enable immune evasion, thereby supporting design strategies that remain effective as the virus evolves (Thadani et al., 2023). Flowing from this, EVE-Vax developed by Youssef et al. (2025) extended the approach toward proactive design, proposing computationally designed antigens that mimic plausible immune-escape trajectories, enabling preclinical evaluation of vaccines against variants that may not yet be dominant (Immunity, 2025). Beyond antigen and epitope design, AI has also been increasingly used in the design and optimization of clinical trials. For example, AstraZeneca leveraged AI-based simulation to evaluate outcomes under different dosing regimens, supporting adaptive trial design decisions (El Arab et al., 2025).

### 3.4 Influenza

Influenza vaccine design is uniquely dependent on forecasting, making it an ideal target for AI approaches that integrate viral evolution, antigenicity, and epidemiological dominance. A key recent example is VaxSeer, an AI-based evolutionary and antigenicity framework developed Shi et al. (Zeng et al., 2021) that has been proposed to rank vaccine strains by prospective “coverage” – a measure that combines predicted antigenic match with predicted future dominance of circulating viruses. VaxSeer leverages protein language model representations and a dynamic dominance predictor to improve prospective selection at scale, directly aligning with the real-world decision bottleneck in seasonal vaccine strain updates (Shi et al., 2025).

Importantly, across SARS-CoV-2, influenza, and other viral diseases, a practical enabler has been the growing use of foundation models for protein structure and design. AlphaFold2 has been broadly adopted for structure inference where experimental structures are unavailable or incomplete, supporting antigen selection and structure-informed epitope mapping (Jumper et al., 2021). Other models such as RoseTTAFold (Baek et al., 2021) have also shown strong structural predictive ability, aiding studies of structural interactions in antigenic proteins and molecular complexes. On the generative side, diffusion and sequence-design models (e.g., RFdiffusion and ProteinMPNN) are accelerating the prototyping of candidate scaffolds, binders, and immunogens, reducing iteration cycles for structure-based vaccine concepts while still typically requiring substantial wet-lab validation (Watson et al., 2023; Dauparas, 2022).

### 3.5 Other viruses

Beyond SARS-CoV-2 and influenza, AI-assisted approaches have also been applied to vaccine research for a range of other viral pathogens. However, unlike COVID-19 and influenza, where multiple AI frameworks have been developed and deployed at scale, most studies in other viral systems do not rely on virus-specific AI models, as they leverage broadly applicable, pathogen-agnostic computational tools that can be transposed across viruses. Notable examples include structure prediction and modelling platforms such as AlphaFold2 and RoseTTAFold (Gao et al., 2025) which are routinely used to infer antigen structures where experimental data are limited, enabling structure-informed epitope mapping and immunogen design across diverse viruses, including hepatitis viruses, mpox (monkeypox), Zika virus, and others. For example, in mpox vaccine research, ML driven immunoinformatics pipelines have been used to prioritize candidate epitopes through the use of pre-trained AI models for antigenicity and immune recognition (Adugna et al., 2025), including MHC binding predictors such as NetMHCpan and NetMHCIIpan, B-cell epitope predictors like BepiPred, and integrated Immune Epitope Database (IEDB) analysis tools, thereby showcasing how broadly applicable AI models can be repurposed to support vaccine target selection for emerging viral pathogens (Adugna et al., 2025). Additionally, a review by Goud et al. described how AI-driven immunoinformatics pipelines employ ML and DL-based epitope prediction models such as NetMHCpan and NetMHCIIpan for T-cell epitope binding prediction, BepiPred for B-cell epitope identification, and AlphaFold2 for structural modelling to support rational vaccine design for avian viral pathogen (Goud et al., 2025).

Beyond these highlighted viral infections and selected tools, numerous ML/AI methods are being incorporated into vaccine design, and Table 1 provides a succinct summary of representative examples.

**TABLE 1:** Summary of existing AI tools for vaccine design.

Tool/Model name	Model type	Primary use case	Sector/Country of origin or use	Data sources used	Strengths	Limitations
Vaxingn-ML (Ong et al., 2021)	Supervised ML-augmented reverse vaccinology care platform	Antigenic and vaccine target predictions	Academic (USA)	Genomic, proteomic, functional annotations	End to end workflows; superior performance in predictive bacterial antigens	No epitopes or structural consideration in prediction models
EvalVax (Liu et al., 2020)	Combinatorial ML	Peptide vaccine design and evaluation	Academic (USA)	SARS-CoV2 genomce sequences, HLA binding and halplotype fquency data	Population level predicted coverage	No immunogenicity assay data sets considered
MUNIS (Wohlwend et al., 2025)	Deep learning with bimodal architecture	Predicting CD8^+^ T-cell epitopes	Academic (USA)	Human Leukocytes antigens ligands and immunopeptidomic datasets (HLA)	Outperforms major existing epitopes prediction tools	Limited HLA coverage, potentially limiting eneralizability across diverse populations
VaxSeer (Shi et al., 2025)	Protein Language Model	Influenca vaccine strain selection support	Academic (USA)	Influenza HA protein and HI datasets; past influenza vaccine effectiveness datasets	Integrates evolutionary forecasting and antigenic match prediction; outperforms WHO strain selection model	Limited antigenic scope, as it is restricted to HA protein only
RoseTTAFold diffusion (Watson et al., 2023)	Diffusion probabilistic model paired with ProteinMPNN	Denovo protein design – binder and oligomer design; active-site scaffolding	Academic (USA)	Protein Data Bank/Experimental Validation Data	General-purpose use for many design segments	Primarily for design and requires wet-lab screening
AlphaFold2 (Jumper et al., 2021; AlphaFold2, 2025; Higgins, 2021)	Deep learning model	Protein 3 structure predictions	Google DeepMind (United Kingdom)	Protein Data Bank, Multiple sequence alignments datasets	Near experimental accuracy and reliable confidence-level estimates that has enabled multiple vaccine designs	Not necessarily capture multistate conformations and weaker performance for intrinsically disordered regions
Epigraph (Epigraph hemagglutinin vaccine induces broad, 2025)	Graph-based computational model	Antigen design for highly diverse virus	Academic and National Institute (USA)	HA sequences and influenza isolates repository	Designs cocktails of antigens that covers common epitopes across viral populations	Does not entirely conformational epitopes and other structural constraints
NetMHCpan (Jurtz et al., 1950; Reynisson et al., 2020)	Artificial neural network	Predict MHC class I antigen presentation; epitope discovery, ranking and prioritization	DTU Health Tech (Denmark)	Over a million binding affinity measurements and mass spectomety eluted ligands; thousands of pathogen-derived epitopes	Supports multi-allelic immunopeptidomics and broad allel and species coverage’ generally accessible	Performance depends on the availability and quality of binding affinity data
Evaxion RAVEN (Using AI to create, 2025)	Pripprietary AI-immunology platform	Rapid identification of viral vaccine candidates	Evaxion Biotech (Denmark)	Genomics, Proteomics and Antigenic pools	Rapid response to emerging viruses and broad inclusion of T-cell components	Not universally accessible as it is proprietary
RiboNN (Nature Biotechnology, 2025)	Deep learning (convolution neural network - CNN)	mRNA translation efficiency prediction to guide mRNA vaccine design	Academic and Industry Collaborator (Sanofi) (USA)	Transcriptomics-wide atlas of mRNA translation efficiency measurements acquired through ribosome profiling	Improved accuracy compared to earlier TE prediction approaches	Not an end-to-end therapeutic performance as it does not validate immunogenicity or efficacy
DeepImmuno (DeepImmuno, 2025)	CNN	Vaccine target selection and insilico peptide design generation	Academic (USA)	Immune Epitope Database	Outperforms most used immunogenicity workflows; highlight salient peptide residues	Scope constraints as it is limited to 9/10-mer peptides

## 4 Existing AI models for antiviral drug discovery and optimization

The current landscape of antiviral drug discovery is being fundamentally reshaped by the integration of computational methodologies, which offer necessary speed and efficiency over traditional time-consuming and costly development pipelines (Kolarič et al., 2026; Mohanty et al., 2020; Namba-Nzanguim et al., 2022). These advanced pipelines heavily leverage deep learning (DL) and machine learning (ML) for several critical functions, ranging from identifying novel targets to optimizing lead compounds.

One established approach involves Quantitative Structure-Activity Relationship (QSAR) modeling, now frequently augmented with AI (Gautam et al., 2025). AI-integrated QSAR is employed for multi-target drug discovery (Kleandrova et al., 2021) and the prediction of potent inhibitors against specific viral proteins, such as the non-structural (NS) proteins of Dengue virus. Furthermore, generative models are used to explore new chemical space and facilitate fragment-based lead optimization, exemplified by models like DeepFrag (Kolarič et al., 2026).

Structure-based drug design (SBDD) is significantly accelerated by ML integration (Prasad and Kumar, 2021). Full SBDD protocols, which typically involve molecular docking and ADME analysis against targets like SARS-CoV-2, Zika, and Hepatitis C virus (HCV) enzymes, are being streamlined (Solo et al., 2023). ML is now routinely combined with molecular docking to enhance the prediction of potential inhibitors; a strategy successfully applied against Dengue virus (Hanson et al., 2024). More sophisticated frameworks integrate molecular dynamics, docking, and ML to predict binding efficacy for key viral enzymes, such as the SARS-CoV-2 Papain-like Protease (PLpro) (Varghese et al., 2025). AI has also been used for virtual screening and hit optimization against the SARS-CoV-2 Spike Receptor-Binding Domain (S-RBD), leading to the identification and synthesis of novel inhibitors (AI Promoted Virtual Screening, 2025).

Crucially, deep learning is instrumental in accelerating drug repurposing and large-scale library screening by identifying latent connections in vast biomedical datasets (Prasad and Kumar, 2021). This application enables the rapid assessment of existing drugs against emerging threats. Recent efforts include using interpretable DL and molecular docking frameworks to repurpose compounds as inhibitors for the SARS-CoV-2 Main Protease (M^Pro^) (Huang et al., 2025). Repurposing is also facilitated by the generation of SARS-CoV-2 protein interaction maps, which highlight druggable human host factors targeted by existing drugs (Gordon et al., 2020). Similarly, attention-based deep learning models have been utilized to repurpose drugs, such as Cangrelor, as potential inhibitors of the Nipah virus RNA-dependent RNA polymerase (Huang et al., 2025). This integration of AI/ML across computational and structure-based methods is key to the modern antiviral discovery pipeline, including the development of covalent broad-spectrum inhibitors for human coronavirus (M^Pro^) (Nature Communications, 2025).

### 4.1 Real-world applications and validation

The translation of AI models from computational predictions to validated therapeutic leads is underscored by numerous real-world applications across various viral families, demonstrating a shift toward systems that influence preclinical and clinical development (Namba-Nzanguim et al., 2022; Varghese et al., 2025).

The rapid response necessitated by the COVID-19 (SARS-CoV-2) pandemic catalyzed some of the most visible successes. AI-driven repurposing efforts were critical in mapping the SARS-CoV-2 protein interactome with human host factors, identifying 66 druggable human proteins targeted by 69 compounds (Gordon et al., 2020). This map accelerated the clinical investigation of drugs like Baricitinib (Mohanty et al., 2020). Advanced AI frameworks, including interpretable deep learning and molecular docking, have been successfully applied to propose existing compounds as inhibitors for the SARS-CoV-2 Main Protease (M^Pro^), providing rapid and specific hit identification (Prasad and Kumar, 2021; Huang et al., 2025). Furthermore, AI facilitated the virtual screening and optimization against the SARS-CoV-2 Spike Receptor-Binding Domain (S-RBD), leading to the synthesis of novel, experimentally validated inhibitors (AI Promoted Virtual Screening, 2025). More recently, AI-supported optimization has led to the discovery of novel, covalent broad-spectrum inhibitors targeting human coronavirus (M^Pro^) (Nature Communications, 2025) and has been used to screen broad-spectrum marine natural products as antiviral agents against coronaviruses (Boswell et al., 2023).

Beyond coronaviruses, AI has a demonstrated impact on historically challenging viral targets. For Human Immunodeficiency Virus (HIV) and Hepatitis C Virus (HCV), machine learning methods are extensively utilized for predicting the emergence of antiviral resistance (Marco, 2025), optimizing combination therapies, and identifying novel drug targets (Kolarič et al., 2026). Specifically for Hepatitis B Virus (HBV), AI is employed for mechanistic understanding, analyzing multi-omics data to pinpoint novel biomarkers and therapeutic targets against the virus’s complex lifecycle (Hu and Jiang, 2025). For HCV, pattern recognition algorithms and deep learning models have been utilized in pharmacogenomics and drug repurposing studies involving agents like Ribavirin and Lopinavir, enhancing the understanding of therapeutic response (Calvo et al., 2025).

The success in broad-spectrum antiviral discovery is a particularly critical area for pandemic preparedness. AI is instrumental in identifying drugs that inhibit essential host factors, a host-directed approach less susceptible to viral mutations. For instance, the Sec61 inhibitor Apratoxin S4, identified through screening, potently inhibits SARS-CoV-2, influenza A virus, and flaviviruses (Zika, West Nile, and Dengue virus) by targeting a host-essential protein (Boswell et al., 2023). Furthermore, models employing multi-view non-negative matrix factorization can predict antiviral drugs against entirely new viruses, effectively addressing the cold-start problem (Su et al., 2022). This allows for the repurposing of drugs, such as Cangrelor against the Nipah virus, confirming the high potential of AI to rapidly accelerate countermeasures against diverse emerging zoonotic threats (Gawande et al., 2025).

### 4.2 Summary of AI tools used in antiviral development

The integration of artificial intelligence (AI) into antiviral development has transitioned from conceptual frameworks to robust, deployment-ready pipelines. This paradigm shift is characterized using diverse modeling strategies tailored to specific stages of the drug discovery lifecycle, ranging from target identification to lead optimization (Kolarič et al., 2026). Quantitative Structure-Activity Relationship (QSAR) models, such as i-DENV, have been instrumental in predicting inhibitors for Dengue virus non-structural proteins by utilizing molecular descriptors and regression-based machine learning (Gautam et al., 2025). Similarly, generative models like DeepFrag and transformer-based architectures allow researchers to explore vast chemical spaces to design *de novo* molecules with optimized pharmacological properties, such as improved binding affinity and LogP (Green et al., 2021; Mao et al., 2024). These tools frequently leverage structural data from the Protein Data Bank (PDB) and chemical libraries like ChEMB(76)L to train deep convolutional and attention-based networks.

Drug repurposing remains a dominant strategy, particularly for emerging threats where “cold start” problems persist. Tools like Constrained Multi-view Nonnegative Matrix Factorization (CMNMF) address this by integrating virus and drug similarity networks to identify potential candidates for SARS-CoV-2 (Su et al., 2022). Furthermore, network-based repurposing engines, such as the SARS-CoV-2 Protein Interaction Map, have identified host-directed targets that successfully influenced clinical trials for drugs like Baricitinib (Mohanty et al., 2020; Gordon et al., 2020). Despite these achievements, significant limitations remain. AI models often struggle with high false-positive rates in virtual screening and require extensive laboratory validation to confirm biological activity (Namba-Nzanguim et al., 2022; Livieratos et al., 2025). Lab validation challenges, particularly in low-resource settings, have led to the development of open-source initiatives like Ersilia, which aim to provide accessible ML models to researchers globally (Turon et al., 2025). As these tools move from computational hits to validated trials, the emphasis is shifting toward interpretable AI frameworks that provide mechanistic insights into drug–target interactions (Huang et al., 2025). These examples are summarised in Table 2.

**TABLE 2:** Summary of AI tools used in antiviral development.

Tool/Model name	Modelling strategy	Target area	Institution/Country	Data types used	Notable outputs/Achievements	Known limitations	Stage of use
i-DENV (Gautam et al., 2025)	QSAR (SVM, ANN)	Viral proteins (NS3, NS5)	CSIR-Institute of Microbial Technology (IMTECH)/India	ChEMBL, DenvInD	Identified Micafungin and Cangrelor as hits	Requires further *in vitro*/*in vivo* validation	Validated hits (computational)
DeepFrag (Green et al., 2021)	Generative (CNN)	Lead optimization	Univ. of Pittsburgh/USA	Structural data (PDB)	Accurate fragment recommendations for ligands	High computational expertise required	Computational only
SARS-CoV-2 Map (Gordon et al., 2020)	Network-based Repurposing	Host pathways	University of California, San Francisco (UCSF) Quantitative Biosciences Institute Coronavirus Research Group (QCRG)/USA	AP-MS, transcriptomes	Identified 69 repurposable drugs (e.g., Baricitinib)	High-confidence PPI does not ensure inhibition	Trial influence
CMNMF (Su et al., 2022)	Matrix Factorization	New viruses (Cold start)	Chinese Academy of Science (CAS)/China	Virus/drug similarities	Effective drug repurposing for SARS-CoV-2	Dependency on multi-view data quality	Computational only
Ersilia Hub (Turon et al., 2025)	ML Platform/Hub	Antivirals (LMIC focus)	Ersilia Initiative/Spain & Africa	Open-source chemical data	Democratized AI access for infectious diseases	Limited infrastructure for lab follow-up	Computational infrastructure
Transformer Model (Mao et al., 2024)	Generative (Transformer)	Broad-spectrum	Bio-IT/South Korea	SMILES, IUPAC names	Controlled molecular generation (LogP)	Readability vs. atomic accuracy	Computational only
AlphaFold (Jumper et al., 2021)	Structure Prediction	Viral proteins	Google DeepMind/UK	PDB, genomic sequences	Breakthrough 3D modeling of unknown proteins	Does not predict ligand binding directly	Foundational tool
Interpretable DL (Huang et al., 2025)	Repurposing (GNN/Docking)	SARS-CoV-2 Mpro	Zhejiang Univ./China	Chemical libraries	Identification of specific Mpro inhibitors	Potential for false positives in screening	Validated hits (computational)

## 5 Existing AI tools for real-world viral monitoring and surveillance

### 5.1 Current AI-driven surveillance tools

Effective health surveillance is foundational to the discipline of public health; it allows the detection of disease, the monitoring of its spread, and the coordination of effective response strategies on both a local and global scale (Idahor et al., 2025). However, traditional surveillance systems are often hindered by manual, slow data collection methods, and restricted data analysis capabilities (Topeola Balkis Awofala, 2024; Li et al., 2025). AI-driven surveillance tools confront these limitations directly, with tools that have been developed to enhance genomic tracking, outbreak detection, epidemiological forecasting, and track real-world vaccine effectiveness (Topeola Balkis Awofala, 2024). Furthermore, AI tools can leverage data previously unavailable to the public health specialist, e.g., social media analysis for outbreak prediction or wearable sensors for early infection prediction (Li et al., 2025). These tools can complement traditional existing methods to provide a more holistic view of disease activity than previously available.

### 5.2 Genomic tracking

Genomic tracking is a central component in disease surveillance, and AI tools are increasingly enhancing its capacity to detect emerging variants, quantifying new variants’ transmission advantage and anticipating immune escape. As a result of advances in other fields, namely, next-generation sequencing (NGS), pathogen genomes can be generated both quickly and cost-effectively. In addition, NGS can show in detail the antimicrobial resistance genes, and phylogenetic relationships of each of these pathogens (Revolutionizing infectious disease surveillance, 2025). When performed at scale, the vast quantity of data generated from these new methods cannot be reliably analysed by traditional analytical methods. However, the use of multiomics technologies, in combination with computational methods can enable automated, and detailed characterization of pathogen evolution and host-pathogen interactions in near real-time, something previously not possible (Revolutionizing infectious disease surveillance, 2025).

One of the more prominent AI-enabled genomic surveillance tools is EVEscape. EVEscape is a model which predicts which viral mutations are most likely to allow immune escape before they occur. The underlying framework of the model combines a deep-learning model which has learnt evolutionary constraint from large protein-sequence datasets, and detailed biological and structural information about the virus (Hie et al., 2024). In this way, EVEscape can make predictions. The model has been demonstrated to be able to anticipate SARS-CoV-2 mutations which were not until later observed in Omicron sublineages (Hie et al., 2021). Similarly, DeepVariant, designed by google, is a neural network that aims to improve the accuracy of variant calling from raw sequencing reads and has now been used extensively in both clinical and public-health genomics infrastructures. The strength of the network is that it can minimize false variant calls and thereby strengthens downstream genomic surveillance (Jumper et al., 2021; Poplin et al., 2018).

Alongside these AI-focused approaches exist computational platforms which demonstrate a vast capability as it relates to genomic tracking and may see future AI integration. An example is the recently developed CoVerage platform - a computational system for viral genomic surveillance. The system continuously analyzes SARS-CoV-2 sequences worldwide and identifies potential variants of interest with increased transmissibility. A retrospective evaluation of the system revealed that CoVerage can identify 88% of WHO designated variants of interests or variants of concern (VOIs/VOCs), it does this with a precision of 79% and recall of 72% (Nature Communications, 2026). Furthermore, it can do this typically more than 2 months before official WHO designation. Another illustration comes from a study in nature which applied a scalable analytical model to over 7.4 million SARS-CoV-2 sequences and attempted to infer the transmission effects of single-nucleotide variants (SNVs). Results demonstrated that even at a frequency of 1%–2% in a population, the model is correctly able to estimate significant transmission advantages (Lee et al., 2025).

### 5.3 Early outbreak detection

Another way AI is transforming viral surveillance is via early outbreak detection. AI-enabled platforms can scan the vast quantities of unstructured online data on social media sites, blogs, news reports and even trends in search queries (Giri and Gupta, 2024; Borda et al., 2022). These platforms work in real-time, and their detection algorithms identify spikes or patterns in data that human analysts might otherwise miss, thereby isolating anomalous health events or unusual clusters. One important challenge which requires addressing is the filtering of misinformation, as these systems combine data from carefully controlled official data and open-source data, e.g., from social media, they may be prone to hallucination (Borda et al., 2022; Brownstein et al., 2008). One example of such a system is HealthMap, which pioneered this approach of early outbreak detection by continuously scraping online media, official reports and other publicly available sources to detect unusual disease events. The system was responsible for identifying one of the earliest signals for the 2009 H1N1 influenza outbreak in Mexico through news parsing (Brownstein et al., 2008; Freifeld et al., 2008). Another example is BlueDot - this platform utilises natural language processing and pattern recognition on both news and airline data to detect and assess outbreak severity. BlueDot’s system, in late 2019, would flag an unusual pneumonia cluster in Wuhan, a week before official alerts about the outbreak, moreover - extrapolating from information on air travel, the system was also able to predict where the outbreak would spread next (Open Source Early Warning, 2025).

### 5.4 Mobility-based forecasting

AI tools have also been applied to disease forecasting by incorporating human mobility data. Susceptible-Infectious-Recovered (SIR) and Susceptible-Exposed-Infectious-Recovered (SEIR) are classic epidemiological models which are used to describe how infectious diseases spread through populations. These mathematical models now form the foundation of many modern AI forecasting systems (Rama et al., 2025). The advantage of AI-enabled systems when compared to a strictly traditional method is the ability of AI to adapt to ever-changing conditions, and the models’ ability to learn complex spatio-temporal patterns from data. Furthermore, the traditional models hold to fixed assumptions and historical parameters - these limitations stifle their ability to adapt during continuously evolving outbreaks (Kaur and Butt, 2025). These systems utilise data from sources such as Google and Apple Mobility Reports, real-time smartphone location reports and public transit usage. Utilising these data, the systems can forecast disease spread with greater accuracy than would be otherwise possible. These insights are subsequently able to help short-term predictions of local increases in outbreaks or hospitalizations - thereby allowing adequate preparation. AI-augmented mobility forecasting models were widely deployed during the COVID-19 pandemic. Systems such as the GLEAM mobility network model, as well as machine-learning frameworks integrating Google and Apple mobility datasets with LSTM or graph neural networks, demonstrated improved short-term case and hospitalisation forecasting accuracy compared with classical SEIR models (Kapoor et al., 2020; Kara, 2021).

### 5.5 Real-world vaccine and antiviral effectiveness assessment

Beyond the use of AI for detecting outbreaks or forecasting epidemic spread - AI tools are also being used to monitor how effective medical and non-medical countermeasures are performing in the real world. This may be achieved by analysing any one of several different datasets: healthcare datasets, electronic health records, vaccination databases, lab results, and even published literature. From the analysed data, these models are then able to make inferences into trends in protection or treatment outcomes.

Models can analyse anonymised EHRs and other hospital records to discern vaccine effectiveness against infection and hospitalization in real time. In other instances, by combining viral genomic data with data on patient outcomes, algorithms are able to discern when a dominant viral variant is compromising the effectiveness of a monoclonal antibody or antiviral drug, as was seen with different Omicron subvariants, in 2021, which showed a reduced susceptibility to the monoclonal antibodies in clinical use at the time (Nature Communications, 2026). In Israel, comprehensive vaccination databases and outcome registries have been combined with ML-based analytics. These systems can show real-time vaccine effectiveness, and or waning immunity to specific viral variants (Nature Communications, 2026; Haas et al., 2021).

### 5.6 Global implementation examples

Around the world, AI-enabled tools have seen widespread integration into both national and international healthcare infrastructures, to bolster public health surveillance and response beyond what is ordinarily possible with traditional methods.

Epidemic Intelligence from Open Sources (EIOS) is the WHO’s approach to integrating AI into public-health decision making (Kim et al., 2025). EIOS aims to improve public health responses by connecting experts around the world and providing them with the best possible tools to detect, assess, and share information. One application of EIOS is the platform’s system for early threat detection - it aggregates reports from websites, news media, social platforms and other open platforms in the original languages continuously and parses it - allowing the identification of any outbreak threats in near real-time (Kim et al., 2025; Medialdea Carrera et al., 2025). The system is currently in use by over 120 member states, and 30 organisations worldwide (Medialdea Carrera et al., 2025). In 2023, the Republic of Korea’s Korea Disease Control and Prevention Agency (KDCA) trialled the EIOS system; from June to October, 425 events were detected globally by the KDCA with eight events being detected early before being officially listed on the WHO’s site, at an average interval of 20 days (Kim et al., 2025). This demonstrates how EIOS can aid in the early detection and early response to public health threats.

Similar tools have also been deployed at the regional, and national levels globally. At the Regional level, the European Union’s European Centre for Disease Prevention and Control (ECDC) have developed and deployed their own tool called ‘Episomer’. Episomer builds on the previous ‘epitweetr’ and focuses on monitoring trends of social media posts by time, place and topic (Espinosa et al., 2022). In the US, the Centers for Disease Control and Prevention (CDC) has already incorporated AI into two of its programs. One of these programs is the National Syndromic Surveillance Program (NSSP) which utilises AI for real-time analysis of patient symptom data from Emergency departments nationwide (CDC, 2025a; CDC, 2025b). Second is FluSight, which utilises AI and ML to forecast future flu activity to help hospitals better plan potential increases in patient number (CDC, 2025b; CDC, 2025c). In both the United Kingdom, and Israel - they can evaluate in near real-time vaccine effectiveness and patient outcomes by using their comprehensive database of primary-care records and vaccination registries. This system allows the detection of waning immunity, and the analysis of vaccine effectiveness on new variants (Haas et al., 2021; CDC, 2025c).

These examples, which are represented in Table 3, show that AI-driven how widespread and diverse AI integration into public health surveillance has become. It is being operationalised across diverse health systems, albeit with variable maturity, data availability, and infrastructural capacity.

**TABLE 3:** Summary of artificial intelligence approaches applied across key stages of vaccine design, highlighting their functions, data requirements, advantages, and current limitations.

Tool	Model category	Location of use	Data streams used	Performance indicators	Strengths	Limitations	Current implementation level
BlueDot (MacIntyre et al., 2023; U of T News, 2025)	Digital epidemiology and global spread forecasting	Global - commercial platform	News reports, airline ticketing data, official health reports	Flagged an unusual pneumonia outbreak in Wuhan on 31 Dec 2019	Integrates diverse data (real-time news + global travel patterns) for early cross-border risk detection. Multiple languages analysed	Not publicly accessible (subscription-only)	Operational – deployed commercially, with adoption by select health authorities and industry clients
HealthMap (Freifeld et al., 2008; Open Source Early Warning, 2025;U of T News, 2025)	Digital epidemiology (open-source outbreak mapping)	Global (public platform via Boston Children’s Hospital)	Online news media, official reports, social media feeds	Automated text classification and filtering with 84% accuracy in integrating outbreak reports, provided early digital signals for various outbreaks (e.g., COVID-19 in late 2019)	Real-time multilingual monitoring with interactive maps; free and open for public health use; machine-learning filters reduce noise in vast open-source data	High volume of data can still include noise/false alarms; depends on internet reports (may miss events in low-connectivity regions)	Operational – in use since 2006, widely used by researchers and health departments worldwide (global public use)
WHO EIOS (Epidemic Intelligence from Open Sources) (Medialdea Carrera et al., 2025; Medialdea Carrera et al., 2025)	Digital epidemiology (event-based surveillance)	Global (WHO platform, accessible to member public health agencies) - Currently in >120 countries	∼12,000 web sources (news, forums, social media) in multiple languages	Employs NLP for entity recognition and priority scoring to tag relevant reports; system capacity ∼40 million items; however	‘One Health’ scope, multi-language automated ingestion; integrated with WHO risk analysis tools	Access restricted to authorized users; requires human review of signals before dissemination	Operational – used within WHO and by partner nations (global consortium platform, not public-facing)
GPHIN (Global Public Health Intelligence Network) (About - GPHIN - Canada.ca, 2025; The Global Public Health Intelligence Network System, 2025)	Digital epidemiology (news analytics with NLP)	Global (based in Canada’s PHAC; feeds into WHO intelligence)	Online news media and reports (5,000–9,000 articles per day) in 10 languages	Upgraded AI system (2018) uses machine translation and NLP to extract events; detected unusual pneumonia in Wuhan on 31 Dec 2019; credited as WHO’s “single most important” source for early outbreak identification	Multilingual, high-volume early warning system with decades of use; proven track record (e.g., first alerts for SARS in 2003 and early signal for COVID-19)	Outputs require expert analyst curation; relies on media reporting (can miss events with limited press)	Operational – established 1997; AI-enhanced version in use (2018 onward) by PHAC and global health security community (internal use system)
Episomer (Espinosa et al., 2022; GitHub, 2026)	Anomaly detection (social media surveillance)	Europe (developed by ECDC; open source for global use)	Social media posts by time, place, and topic	Monitors social media posts for spikes in health-related terms; flags “unusual” increases by time and location as potential outbreak signals (user can filter by geolocation)	Real-time capture of public concern and symptoms (including informal signals); fully open-source and user-customizable tool	Coverage limited to populations active on social media (bias); high noise-to-signal ratio – requires careful tuning to avoid false alarms; depends on social media sites’ API access and data quality	Operational - open source and accessible via github
NSSP (National Syndromic Surveillance Program) (CDC, 2025a; CDC, 2025b)	Anomaly detection (syndromic surveillance)	USA (nationwide program across state and local health departments)	Emergency department and urgent care visit data (near-real-time patient chief complaints and symptom syndromes)	ML-driven algorithms analyse ∼70% of U.S. ED visits in real time to spot unusual upticks (e.g., clusters of respiratory illness); has improved outbreak detection speed and situational awareness, enabling faster response to events (e.g., surges in flu or novel outbreaks)	Broad health system coverage provides early signals before lab confirmations; continuously monitors diverse syndromes (respiratory, febrile, gastrointestinal, etc.); proven useful for early detection of seasonal flu and other emerging health threats	May generate false alarms from non-infectious events or data noise; quality and timeliness of data depend on hospital reporting; identifying specific cause requires follow-up (syndromic signals are preliminary)	Operational – fully rolled out nationwide (regularly used by CDC and state health departments); ongoing enhancements to incorporate more AI for detection
FluSight (CDC Influenza Forecasting) (CDC, 2025a; CDC, 2025b; CDC, 2025c)	Forecasting (seasonal flu)	USA (national collaboration: CDC with academic and private teams)	Historical flu surveillance data (e.g., ILINet clinic visit rates, lab-confirmed hospitalization rates), plus external indicators (some models use weather, search trends, etc.)	Produces probabilistic weekly forecasts of flu activity and timing; Weighted Interval Score), predicting peaks several weeks in advance	Leverages diverse models (including AI/ML approaches) to create robust ensemble forecasts; openly shared and evaluated forecasts improve planning for vaccination campaigns, staffing, and resource allocation	Forecast accuracy varies by season; performance drops during novel strain emergence or unusual dynamics; typically limited to short-term (4-week) forecasts with uncertainty growing for longer horizons	Operational – annual forecasting since 2013; used by CDC and health officials for winter preparedness (national implementation with ongoing model competitions)
Biobot Analytics (Wastewater Surveillance) (Global COVID-19, 2023; Biobot.io, 2022)	Anomaly detection (wastewater-based)	USA (city and county pilot programs; expanding toward national coverage)	Wastewater samples from sewage treatment plants (tested for viral RNA like SARS-CoV-2, polio, etc.), combined with population and GIS data	Wastewater viral levels provide a leading indicator of community infection trends (independent of clinical testing); AI-powered analytics map results on a real-time dashboard and predict spread patterns from sewage data	Captures asymptomatic and untested infections (broad population coverage); unaffected by testing availability or healthcare-seeking behavior; can quantify infection prevalence in near real-time, enabling early interventions (often several days to weeks ahead of case surges)	Quantitative interpretation can be challenging (viral shedding rates vary; environmental factors affect concentrations); requires laboratory infrastructure and sampling logistics; currently complements rather than replaces traditional case surveillance	Pilot/Scaling – launched during COVID-19 (2020) with >400 sites across 42 states participating; now expanding for ongoing monitoring of multiple viruses (operational pilots moving toward wider adoption in public health systems)
Nextstrain (Hadfield et al., 2018)	Genomic analysis (real-time phylodynamics)	Global (open platform used by worldwide genomic surveillance networks)	Viral genomic sequences shared by labs (via GISAID, GenBank, etc.), with metadata (sample date, location)	Provides near real-time tracking of pathogen evolution and spread via phylogenetic trees and maps. During COVID-19, Nextstrain visualizations of SARS-CoV-2 variants enabled rapid identification of emerging strains and their global distribution (often within days of sequences being uploaded)	Open-access, interactive platform integrating sequence data with geographic and temporal context; fast updates, widely adopted by scientists and public health agencies	Dependent on timely genome sharing (gaps where sequencing capacity is limited); not a predictive tool, interpreting phylogenetic data requires expert knowledge	Operational – core component of global viral genomic surveillance (used by CDC, WHO, and national laboratories for real-time variant tracking); continuously maintained and extended to multiple pathogens (e.g., flu, Ebola, RSV)

## 6 Integrating AI across vaccine design, antiviral discovery, and surveillance

### 6.1 How to connect the three pipelines step-by-step

**FIGURE 1:**
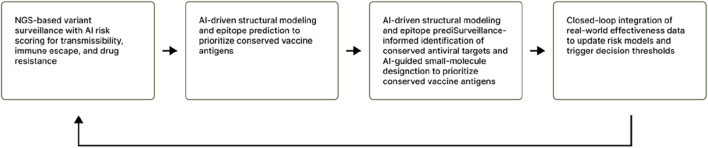
Flowchart illustrating an AI-powered, closed-loop system for variant surveillance, vaccine development, and real-world effectiveness integration to enhance decision-making in pandemic preparedness and response.

To merge the concept of the discovery of antivirus together with the design of vaccines and genomic surveillance into single and uninterrupted system of learning, there is a requirement for the implementation of both governance and technical architecture which will allow for real-time flow of data, adaptive decision making and cross-model compatibility (Fair Principles, 2025). The technical backbone for achieving this is real-time sequencing and metadata capture, this involves the uploading of genomes together with major epidemiological details (such as status of vaccination, location, collection date, clinical severity) to the repositories (either federal or central) in a fixed format to simplify the process of data consumption by AI models (Wilkinson et al., 2016; Wittig et al., 2017). Also, adherence to several data handling principles (inclusive of FAIR principles and metadata standards) facilitates reusability, creates improved findability and its utility across pipelines (Fair Principles, 2025). The steps are explained below, and illustrated in Figure 1.

### 6.2 Step 1: (Surveillance ingestion and variant risk scoring)

This involves the production of raw reads from the Next-generation sequencing pipelines (NGS) which undergo quality checks and are then assembled into genomes (Meijers et al., 2025; Jingzhi, 2024). There is an assignment of a heightened score for transmissibility, resistance to drugs or immunity escape for every new variant by machine learning models (which have been trained on the history of past mutations) (Meijers et al., 2025; Predictive evolutionary modelling for influenza). These risk scoring are to be probabilistic as well as being timestamped to supply product (drug and vaccine) developers with a list of variants that are already ranked deserving of further structural assessment or antigenic (Meijers et al., 2025).

### 6.3 Step 2: (Structure and epitope analysis for vaccine design)

Sequences are being converted into three-dimensional predictions by structural models (such as AlphaFold) for the enablement of design of antigen and mapping of epitopes (Jingzhi, 2024). Also, the prediction of epitopes of B- and T-cell is performed by protein language models and message-passing neural networks, areas representing low tolerance for mutation are flagged for efficient vaccine targets (Iyede et al., 2023; Joseph et al., 2022). Prioritization of antigens for validation of experiments is achieved through the combination of the epitope’s predictions with immune-escape risk scores already derived from the surveillance step above (Jumper et al., 2021; Hie et al., 2024).

### 6.4 Step 3: (Antiviral target identification and small-molecule design)

Functional but conserved residues which are still essential across various variants are being identified by the surveillance process, the combination of these sites with the predicted three-dimensional structures leads to the revelation of druggable pockets. The models of generative chemistry and engines of reinforcement learning can perform the proposal of the utilized lead compounds for achieving resistance robustness and potency, potential liabilities of safety are being screened out by *in silico* ADMET (Das, 2025; Stokes et al., 2020). The candidates that survived are directly fed into the speedy *in-vitro* pipelines for screening (Stokes et al., 2020).

### 6.5 Step 4: (Integrated feedback loops and decision thresholds)

Updating fitness estimation and the probabilities of resistance emergence is achieved by reuploading the clinical effectiveness data (such as rate of vaccine breakthrough, failures of antiviral treatments) into the surveillance model (Zwart et al., 2023). With the presence of this closed-loop system, updates of vaccine strains as well as optimization of iterative antivirus and public health interventions prioritization are pre-empted (Schulze et al., 2023). In practice, there is a need for dashboards which show the confidence bands of each model, the predefined thresholds for alerts and decision logs to be made available to regulators (Jumper et al., 2021).

Swapping of epitope, variants and drug modules by institutions (without having to share the raw data) is made possible by federated learning, JSON/Protobuf ontology and modular micro-services (Rieke et al., 2020; Teo et al., 2024). The creation of government frameworks (such as pre-agreed thresholds for evidence) will ensure the allowance of the use of auditable and transparent AI outputs in decisions that are time sensitive (Rieke et al., 2020).

### 6.6 Operational guidance for researchers and program developers

To put integrated AI pipelines into operations, there is a need for creating steps that are solid and traceable with focus on governance of data, model development, validation as well as downstream validation. The first step includes the establishment of a framework for data governance which demands metadata standards, tracking of data provenance as well as version control, with the inclusion of FAIR principles (Wittig et al., 2017) and the formal agreement for data sharing which has initially indicated the allowed use, period of retention and privacy protection. Implementation of automated ETL pipelines should be adopted by the program for the harmonization of data from lab assay, clinical outcomes and surveillance into shared data schemes (Fair Principles, 2025).

The second step includes building of modular and validated AI components, this includes breaking down of the end-to-end system into validated modules, namely,: “variant detection and risk scoring”, “epitope prediction”, “design of *in silico* drug” and finally “prediction of clinical outcome”. For each module, there is a requirement for the provision of a documented dataset for training purposes, performance indicators (such as AUC and calibration) and a bias assessment system across various population subgroups (Kelly et al., 2019). There should also be the use of model cards together with comprehensive documentation for each of the components for the promotion of transparency and regulatory review (Springer Nature Link, 2025).

The third step of the process is the deploration of federated learning along with safe multi-party computation in situations where it is not feasible to centralize data. This is achieved by training models locally at every site with the privacy of the data still intact as there is an aggregation of model updates centrally which limits the necessity for sharing the patient data (Rieke et al., 2020; Teo et al., 2024). The usefulness of this approach is particularly seen in global surveillance through several countries operating diverse privacy laws, also, to practically deploy this approach, there is a need to have orchestrators (like TensorFlow Federated) and solid protocols for cryptography (Rieke et al., 2020).

The fourth step includes embedding validation pipelines and continuous monitoring, one of the major risks of the process is post-development model drift (Springer Nature Link, 2025). This is caused by the degradation of predictive performance because of pathogen evolution. Hence, implementing a standard continuous evaluation of the process with the use of prospective cohorts in combination with data from monitoring dashboards (performance indicators, calibration plots, etc.) is important (Springer Nature Link, 2025).

The fifth step involves the construction of inter-disciplinary governance and pathways for decision-making, there must be a pre-definition of the thresholds for action triggers (like starting an antiviral pivot) by public health authorities, regulators and researchers. There should be an incorporation of model uncertainty with health impact modelling by the thresholds, also, compositions of the decision-making boards should include representatives of the community (Jingzhi, 2024).

The last step involves capacity building and development of the workforce, there should be an upskilling process for several medical workers (such as clinicians, immunologists) in AI content by the training programs, also, data scientists should be trained in the context of biology (Goud et al., 2025; Author Anonymous, 2024).

### 6.7 Concrete integration examples

There are various examples of research and operations that demonstrate how the design of vaccine, surveillance and antiviral discovery are integrated.

The first set of examples illustrate the real-time surveillance of variants which informs the design of vaccines. Ranking of strains for vaccine updates can be achieved by the application of computational frameworks that allows for the combination of antigen cartography with neutralization assays and phylogenetics (Jingzhi, 2024; Krammer, 2020). There are promises shown by predictive models in the substitution forecast of SARS-CoV-2 and dominant influenza; this has facilitated the pre-emptive selection of strains of vaccines in simulation research (Meijers et al., 2025; Jingzhi, 2024). Also, there is an integration of genomic dashboards in early operations with selection meetings for antigens by manufacturers to reduce the duration between variant detection and synthesis of antigen (Jingzhi, 2024).

The second set of examples illustrates the prioritization of antiviral targets from surveillance data; conserved and functional domains can be brought to light when subjected to solid purifying selection (proper targets for antivirus) using longitudinal genomic surveillance (Krammer, 2020). Proposals of molecules for binding conserved pockets have been made with chemogenomic screens that are driven by AI (predictors of activities and comprehensive generative models), validation of candidates was swifter in the wet-lab screenings afterwards when compared to the traditional screenings (Das, 2025; Stokes et al., 2020). The third set of examples itemizes joint dashboards whereby treatment response is linked with evolution of virus and effectiveness of vaccine. The combination of neutralization data and effectiveness of vaccines has given room for the estimation of relative effectiveness by regulators with respect to the developing variants, also, prioritization of booster formulations is employed (Schulze et al., 2023). There must be an implementation of cautious adjustment for bias by these platforms to prevent the occurrence of false signals (Anjaria et al., 2023).

One of the most mature use cases is real time variant surveillance informing next-generation vaccine redesign, as exemplified by the VaxSeer framework. VaxSeer selects vaccine strains based on a predicted coverage score generated through an *in silico* pipeline that integrates viral surveillance and antigenic prediction. Given circulating viral proteins and candidate vaccine proteins, a dominance predictor estimates the expected prevalence of each viral protein in the upcoming season, while an antigenicity predictor uses pairwise sequence alignment to forecast hemagglutination inhibition (HI) test results. These outputs are combined to compute a predicted coverage score for each vaccine candidate by averaging predicted antigenicity across circulating viruses, weighted by their predicted dominance (Shi et al., 2025). This approach directly integrates genomic surveillance data, such as sequences deposited in GISAID, with antigenicity data derived from WHO Collaborating Centre HI assays, thereby operationalizing real time surveillance into vaccine strain selection rather than post hoc evaluation. The same surveillance driven logic extends beyond vaccine strain selection into antiviral and immunogen target identification based on mutation patterns.

High infectivity combined with rapid mutation rates, as observed in SARS-CoV-2, has motivated AI driven models capable of predicting mutational landscapes and immune escape. NLP based algorithms developed by Hie, a computational biologist and AI researcher with his team, can predict mutations that preserve pathogenicity while enabling immune escape, revealing structural escape patterns across viruses including SARS-CoV-2. Related approaches using neural networks and rough set theory have demonstrated the ability to predict nucleotide changes in successive viral generations, while large scale analyses of thousands of SARS-CoV-2 sequences have enabled the identification of epitope hotspots suitable for broad spectrum vaccine and antiviral design. Recurrent neural network-based LSTM models and phylogeny informed reverse engineering approaches further demonstrate how surveillance derived mutation data can be transformed into forward looking predictions of viral evolution (Arora et al., 2021).

Together, these systems illustrate how AI pipelines link real world mutation surveillance directly to antiviral target prioritization. The use of AI approach in a structured way can help ensure precise identification of antigens and relevant epitope through models like the AlphaFold2 that can generate error free three-dimensional models of viral proteins and revealing conformational epitopes that cannot be determined from just linear sequence data. To ensure precise predictions, pLDDT scores and Ramachandran plot analyses helps to facilitate the identification of surface-exposed and conserved regions with functional relevance for immune recognition.

All of these are combined with tools such as BepiPred, ABCPred, DiscoTope, NetMHCpan, and NetMHCIIpan, within multi-stage filtering frameworks that account for antigenicity, allergenicity, toxicity, and population coverage. In applying these pipelines, across multiple viral pathogens, including influenza viruses, infectious bronchitis virus, Newcastle disease virus, IBDV, CAV, and fowl pox virus, it allows for their generalizability and scalability as surveillance-informed platforms for vaccine and antiviral target discovery (Goud et al., 2025). These capabilities naturally support dashboard style systems that combine surveillance data, predicted antigenic drift, therapeutic performance, and vaccine effectiveness metrics, transforming fragmented datasets into actionable, real time situational awareness (Goud et al., 2025). Taken together, these examples show that integrated AI pipelines are already being tested or proposed across the full continuum of viral threat response from real time variant surveillance and mutation informed antiviral discovery to population aware vaccine design and outcome linked monitoring systems. Although potential implementation is illustrated by integration, there are several practical limitations which include gaps in the standardization of data, generalizing of data across several populations and inequality in the sequencing capacity across the globe. To overcome these limitations, there is a need for investments to be made in sequencing networks worldwide, federated analysis as well as transparent models for practice validation also require proper investment (npj, 2025).

## 7 Future directions and implementation considerations

Artificial intelligence possesses diverse applications in the antiviral ecosystem from vaccine design to real-time surveillance. However, there are significant challenges faced regarding implementation and scalability, especially in low and middle-income countries as highlighted by the World Health Organization (Global strategy on digital health, 2025). For instance, the absence of readily available digital medical and health records in certain geographic settings may create gaps in demographic and clinical data which are primary inputs for training AI and ML models (Wahl et al., 2018). The effectiveness of vaccine or antiviral drug designs using AI also depends on the availability of large, diverse, quality datasets. A crucial concern revolves around algorithmic bias using data that may be skewed or not representative of target populations (Ghosh et al., 2023). Now, most AI/ML tools available to the research community have been trained on historical (public) data collected from large chemical and bioactivity databases, as well as ‘omics’ resources and biomedical knowledge bases. Therefore, the availability and performance of AI/ML models are biased largely towards areas that have traditionally received more attention and for which richer datasets are consequently available. Infectious disease research is hampered by the lack of validated targets, poor molecular characterization of the pathogens and scarcity of large screening datasets (De Rycker et al., 2018). Hence, AI-enabled antiviral discoveries may suffer contextual bias because of extensive concentrated developments and deployment in high-income countries. This paradox allows for models to be potentially trained with wrong data, fostering inequality and counters the decentralization of AI tools in low-resource settings (Weissglass, 2022). Biased algorithms can then affect predictions and impact the safety and efficacy of AI-designed vaccines (Carr, 2023). Ensuring privacy and security of large datasets used to train AI models is as important as addressing algorithmic biases that could lead to disparities in access to antiviral therapeutics (Ghosh et al., 2023). Regulatory bodies such as the Food and Drug Administration are actively considering the use of AI in decision-making for drugs and biological products with emphasis on validation and safety evaluations (Artificial Intelligence for Drug Development, 2025). Besides validation, explainability, interpretability, risk mitigation, consent, and data privacy are other ethical and regulatory pillars guiding AI decisions in medical and public health settings (Rodríguez-González et al., 2019). Explainability and interpretability are critical bottlenecks that drive fair and transparent algorithms, as a lot of AI models, especially deep learning architectures are challenging to decipher and understand (Rodríguez-González et al., 2019). For AI-designed antivirals, ensuring safety through pre-clinical studies and clinical trials, as with their traditional counterparts may be required (Ghosh et al., 2023). The development and deployment of automated technologies powered by AI in vaccine design, drug discovery and real-time surveillance must be guided by ethical principles of beneficence, non-maleficence, justice and autonomy (Springer, 2025). Concerning viral epidemiological surveillance and monitoring, a potential pitfall of artificial intelligence is the generation of false positive or false negative test results. AI-based systems may identify patterns unrelated to disease outbreaks or miss important signals due to algorithm and data limitations (Anjaria et al., 2023).

### 7.1 Building resilient AI-enabled antiviral ecosystems

The creation of adaptive and resilient antiviral ecosystems may demand that AI-based models be considered for training on well-developed legal and regulatory frameworks tailored to health systems’ needs of target communities, especially LMICs. This will pave the way for careful adoption and prevent health disparities in an era of emerging and endemic viral threats (Artificial Intelligence, 2025). Due to the participation of AI in different applications, there may also be a need for ethical governance to handle safety and liability concerns connected to vaccine design, drug discovery and surveillance. Ethical auditing can examine the inputs and outputs of AI algorithms and models for bias and potential risks (Cath, 2018). Regulatory bodies also could collaborate to create a harmonized environment without compromising safety and ethical standards. This may involve unified regulatory frameworks that facilitate the smooth integration of AI technologies into vaccine development, drug development and surveillance processes. Local regulation and legal frameworks, strategies, and policies are fundamental to successfully deploying AI solutions. The regulation includes the provision of privacy, security, informed consent, ethics, liability, confidentiality, trust, equity, and accountability policies. Due to ethical considerations, developing robust protocols for data sharing that prioritize privacy and confidentiality is essential to mitigate ethical concerns related to patient data, alongside clear ethical guidance covering additional data privacy, informed consent, and data sharing (Hare et al., 2024).

Despite efforts by the scientific community to collect experimental data on anti-infective molecules, scarcity of publicly available data in diseases of interest such as antivirals hinders the development of novel AI/ML tools. A strategic avenue to overcome this limitation is to leverage the knowledge accumulated over the years by pharmaceutical companies. While pharmaceutical companies publish their results in scientific publications, only a small subset of the molecules is screened to protect the industry’s intellectual property (IP). Incomplete disclosure of these experiments deters the full realization of data-driven drug discovery (Mervin et al., 2015). Data-sharing techniques using privacy-preserving AI/ML approach proposes that IP-sensitive data can be effectively made available in the form of AI/ML models, which retain the essential properties of the training data but do not reveal the identity of the compounds used to train the model. A classic example of this is the MELLODDY consortium which orchestrates data-sharing between ten pharmaceutical companies while decentralizing data and preventing exposure to proprietary information (Burki, 2019). This creates an environment for rapid therapeutic updates and bridges the data gap behind AI-based drug discoveries.

**FIGURE 2:**
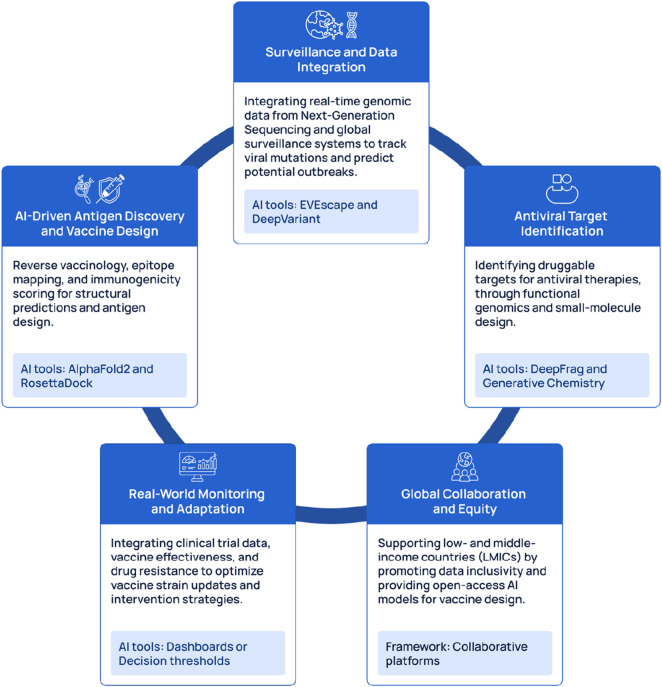
Future directions and implementation strategies in vaccine design with AI.

The existing limitations of AI-based surveillance systems particularly in reference to false test results underscore the need for sustained oversight and investments in data science and ethics training for public health officials to guarantee responsible use of these systems (Anjaria et al., 2023). Despite many advancements, the integration of AI with traditional surveillance faces obstacles such as interoperability issues, data-sharing constraints, and varying levels of technological infrastructure across countries (Ameh, 2024). Standardized protocols for AI implementation, fostering international collaboration, and ensuring that AI tools remain interpretable for epidemiologists and policymakers will be essential for their long-term success (Udegbe et al., 2023). By leveraging AI’s computational power while maintaining the rigor of traditional epidemiological methods, public health authorities can enhance their capacity to predict, monitor, and respond to pandemics more effectively than ever before (Olaboye et al., 2024). Figure 2 below summarizes this section.

## 8 Recommendations and conclusion

### 8.1 Recommendations

Future work in AI-driven vaccine design should place greater emphasis on the quality, diversity, and biological relevance of the data used to train computational models. Many existing AI frameworks rely heavily on genomic and immunological datasets generated in high-income settings, which limits their applicability to populations that are often most affected by infectious disease outbreaks. Expanding training datasets to include viral genomic diversity, host immune responses, and clinical outcome data from low- and middle-income countries will improve the generalisability and equity of AI-derived vaccine candidates. In addition, greater standardisation in data collection, annotation, and sharing across research groups will be essential to reduce bias, enhance reproducibility, and allow meaningful comparison between AI models.

There is also a need to strengthen the integration of AI tools within traditional vaccine development pipelines. AI-generated predictions of epitopes, antigen structures, and immune responses should be systematically linked to experimental validation using *in vitro* assays, animal models, and early-phase clinical studies. Close collaboration between computational scientists, immunologists, clinicians, and regulatory experts will be critical to ensure that AI outputs are biologically interpretable and clinically actionable. Finally, ethical and governance considerations should be addressed early in the development process, including transparency of algorithms, accountability for model decisions, and equitable access to AI-enabled vaccine innovations. Addressing these issues will help ensure that advances in AI contribute meaningfully to safe, effective, and globally accessible vaccines.

## 9 Conclusion

Artificial intelligence is increasingly influencing how vaccines are conceptualised, designed, and optimised, offering powerful tools to accelerate responses to emerging and re-emerging infectious diseases. By enabling rapid analysis of large-scale genomic, structural, and immunological data, AI has the potential to reduce development timelines and improve the precision of antigen selection. However, the successful application of AI in vaccine design depends not only on computational advances but also on the availability of high-quality, representative data and strong links to experimental and clinical validation. Without careful integration into established biological and regulatory frameworks, AI-driven approaches risk producing outputs that are difficult to translate into real-world impact.

As the field matures, the greatest gains will come from interdisciplinary collaboration and responsible innovation. Ensuring transparency, addressing algorithmic bias, and prioritising equitable access will be essential if AI-enabled vaccine development is to benefit populations globally, rather than reinforcing existing disparities. With sustained investment in both computational methodologies and translational research, AI has the potential to complement and strengthen conventional vaccine development, contributing meaningfully to global health preparedness and future pandemic response.
